# Distal 10q trisomy with copy number gain in chromosome region 10q23.1–10q25.1: the Wnt signaling pathway is the most pertinent to the gene content in the region of copy number gain: a case report

**DOI:** 10.1186/s13104-015-1213-x

**Published:** 2015-06-19

**Authors:** Siew-Lee Wong, Hsin-Hsu Chou, Chung-Nun Chao, Joseph Hang Leung, Yu-Hsin Chen, Cheng-Da Hsu

**Affiliations:** Departments of Pediatrics, Ditmanson Medical Foundation Chia-Yi Christian Hospital, Chiayi, Taiwan; Departments of Radiology, Ditmanson Medical Foundation Chia-Yi Christian Hospital, Chiayi, Taiwan; Departments of Medical Research, Ditmanson Medical Foundation Chia-Yi Christian Hospital, 539 Zhongxiao Road, East District, Chiayi, 600 Taiwan

**Keywords:** Distal 10q trisomy, Chromosome 10q23.1–10q25.1, Array-comparative genomic hybridization, Copy number variation, Copy number variation region, Database for annotation, visualization and integrated discovery (DAVID), GO analysis, KEGG pathway analysis, Wnt signaling pathway, *WNT8B*, *LZTS2*, *NFKB2*, *PTEN*

## Abstract

**Background:**

Complete or partial trisomy 10q involves a duplication of 10q, or the long arm of chromosome 10. Distal 10q trisomy is a well-recognized and defined but rare genetic syndrome in which duplication of distal segments of 10q results in a pattern of malformations. Although abnormal chromosome phenotypes are commonly detected by visualization of chromosomes by traditional cytogenetic techniques, this approach is marginal in both diagnostic sensitivity and potential for biological interpretation, thus making implementation of advanced techniques and analysis methods an important consideration in a health service.

**Case presentation:**

The present study describes the case of a Taiwanese boy from healthy parents with mental, growth, and psychomotor retardations. Additional clinical features included facial dysmorphism, microcephaly, brain atrophy, camptodactyly, and—as the first reported case—bilateral renal atrophy with chronic kidney disease stage 2 and the presence of a renal cyst in one kidney. A novel 21.8 Mb copy number variation region in chromosome region 10q23.1–10q25.1 was verified by array-comparative genomic hybridization in combination with quantitative real-time polymerase chain reaction. Subsequently, 200 protein-coding genes were identified in this copy number variation region and analyzed for their biological meaning using the database for annotation, visualization and integrated discovery.

**Conclusion:**

According to the result of gene functional enrichment analysis using database for annotation, visualization and integrated discovery, the Wnt signaling pathway is the most pertinent to the gene content in the copy number variation region. A change in the expression levels of some Wnt signaling pathway components and of *NFKB2* and *PTEN* genes due to a gain in their gene copy number may be associated with the patient’s clinical outcomes including brain atrophy, bilateral renal atrophy with chronic kidney disease stage 2, a renal cyst in one kidney, and growth retardation.

**Electronic supplementary material:**

The online version of this article (doi:10.1186/s13104-015-1213-x) contains supplementary material, which is available to authorized users.

## Background

Partial trisomy of the long (q) arm of chromosome 10 was first reported in 1965 [1], and since then over 30 reports published have described this genetic disorder that appeared in children. The distal 10q trisomy syndrome, which is well recognized and defined to distinguish it from other forms of partial trisomy, is a very rare chromosomal disorder with the distal portion of the q arm of chromosome 10 appearing three times (trisomy) rather than twice in normal cells of the body. Typical features of this disorder consist of prenatal and postnatal growth retardation, hypotonia, mild to severe mental retardation, and mild to severe psychomotor retardation [2]. Affected individuals may have a distinctive dysmorphic appearance of the head and facial area; abnormalities of the hands and/or feet; and/or skeletal, cardiac, renal, and/or pulmonary defects [2]. The range and severity of symptoms vary from case to case, depending upon the span and location of the duplicated portion of chromosome 10q.

The diagnosis, treatment, and overall management of patients with 10q trisomy will substantially benefit from the identification of the patients’ novel chromosomal disorders and of molecular players involved in the clinical abnormalities, since secondary genetic alterations are usually correlated with patients’ outcomes. In this case study, we first performed chromosome analysis of peripheral blood lymphocytes from the patient. As generally perceived, the traditional cytogenetic method is limited to obtaining results of gross structural abnormalities, and the method revealed no detectable abnormal karyotype in this case. Afterward, we investigated the use of DNA copy number analysis as a tool to identify chromosomal breakpoints important to the biology of the patient’s clinical outcomes. Because microarray-based comparative genomic hybridization (array-CGH) has been described as a robust and cost-effective alternative to traditional cytogenetic methodology in a previous paper [3], we performed whole-genome array-CGH in an attempt to detect genome-wide copy number variations (CNVs) in the patient’s blood DNA sample, followed by validation by quantitative real-time polymerase chain reaction (qPCR). We also performed a systematic integrative analysis of the protein-coding genes identified within the copy number variation region (CNVR) using the database for annotation, visualization and integrated discovery (DAVID) in order to extract the genes’ biological features or meaning, which might be associated with the patient’s clinical features.

## Case presentation

**Figure 1:**
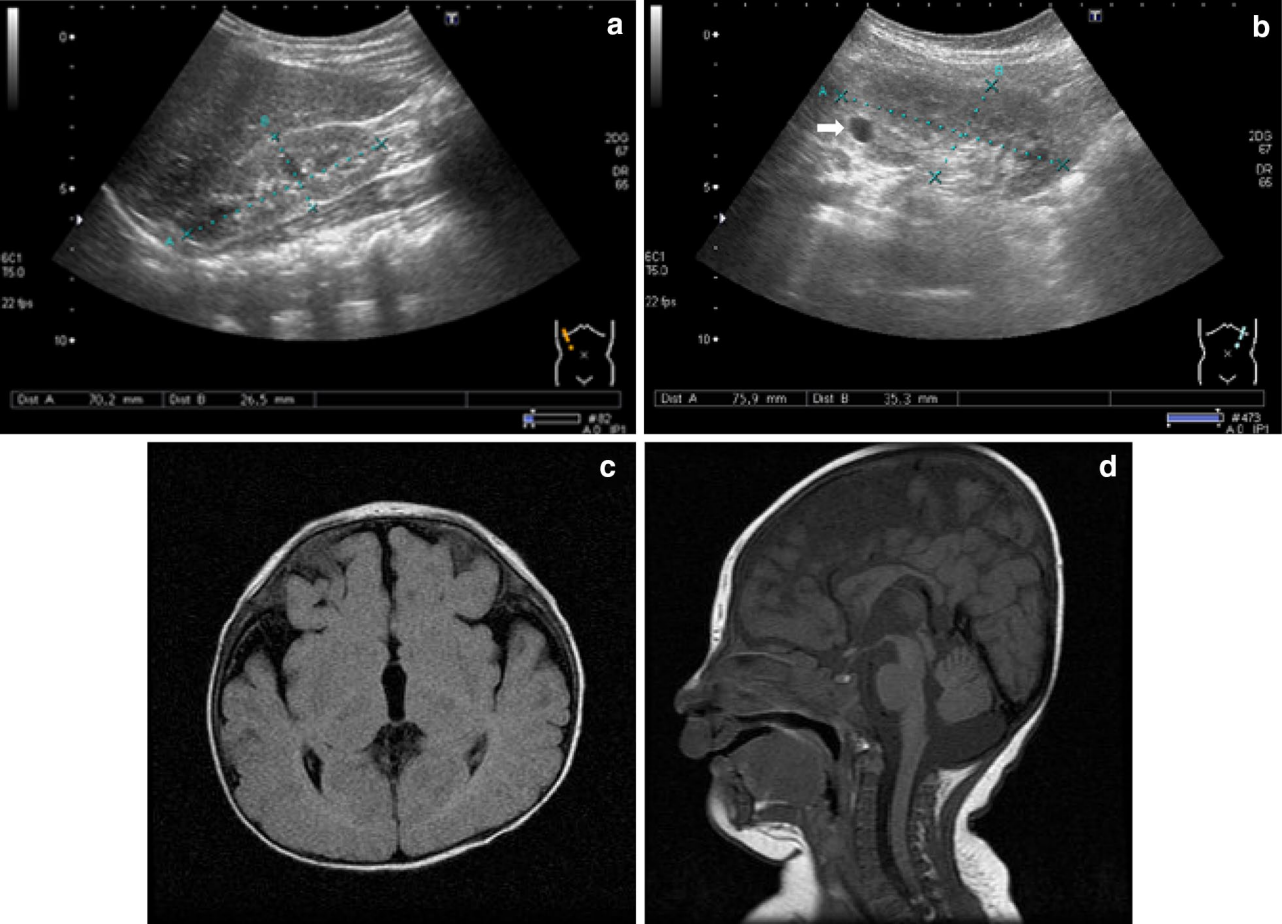
Ultrasonographic and magnetic resonance imaging examinations at 9 years and 4 months of age. Renal ultrasound showed the small size of the kidneys for the patient’s age: **a** the right kidney (length, 70.2 mm) and **b** the left kidney (length, 75.9 mm) with a simple renal cyst (1.13 × 0.78 cm) indicated by an *arrowhead*. Magnetic resonance imaging revealed brain atrophy; **c** diffuse enlargement of the subarachnoid spaces, bilateral widening of the sylvian fissures, and hypomyelination of the anterior white matter; and **d** thinning of the corpus callosum.

The Taiwanese patient was the only child of healthy, non-consanguineous parents, born at term after an uneventful pregnancy. His birth measurements were low for gestational age: weight, 2,025 g (<3rd percentile); height, 45.6 cm (<3rd percentile); and head circumference, 31 cm (3rd percentile). Apgar scores were 9 at 1 min and 10 at 5 min. There were no repeated miscarriages, mental retardation, or malformation syndromes in the family history. He was referred for endocrinological evaluation at 4 years and 10 months of age due to short stature and underweight; his height was 99.7 cm (3rd percentile), weight was 12.8 kg (<3rd percentile), and head circumference was 43 cm (<3rd percentile). Physical examination revealed dysmorphic features including microcephalus, a high forehead, drooping eyelids, short palpebral fissures, a bow-shaped mouth, malformed, posteriorly rotated ears, and camptodactyly. He also had left-sided inguinal hernia and received herniorrhaphy. Delayed psychomotor development milestones were noted. Hearing test results were normal. His bone age was 4½ years. The results of a hormone survey, including thyroid function and insulin-like growth factor 1 tests and tandem mass spectrometry (MS/MS) evaluation, were normal. Echocardiographic findings were nonspecific, and ultrasound examination showed that the size of the kidneys was small for his age (Figure 1a, b). A magnetic resonance imaging (MRI) scan of the brain revealed brain atrophy (Figure 1c, d). Chromosome analysis of peripheral blood lymphocytes showed a normal male karyotype. At the age of 9 years and 4 months, the patient received his individual intelligence test (WISC-IV), which revealed moderate mental retardation with a full scale score of 49. Repeated renal ultrasound examination showed bilateral renal atrophy with chronic kidney disease stage 2 (SCr: 0.56 mg/dL; 24 h CCr: 84.8 mL/min/1.73 m^2^), and a simple renal cyst (1.13 × 0.78 cm) was incidentally identified in the left kidney (Figure 1b). Other internal organs were normal. Subsequently, a molecular examination by array-CGH was performed to identify genetic breakpoints important to the biology of the patient’s clinical outcomes.

### Methods for array-comparative genomic hybridization analysis

#### DNA extraction and array

Genomic DNA was extracted from the peripheral blood of the patient and of a healthy male control. After spectrometric measurement of DNA concentration, the DNA samples were stored at −20°C until used. Array-CGH was performed by Sofiva Genomics Laboratory (Taipei, Taiwan) using the CytoChip Oligo array (BlueGnome, Cambridge, UK) according to the suggestions from the International Standard Cytogenomic Array Consortium (ISCA). The array contained more than 60,000 oligonucleotide probes, which covered the 22 autosomes and 2 sex chromosomes with an average probe spatial resolution of approximately 60 kb and comprised coding and non-coding human genome sequences sourced from the UCSC hg19 human genome (GRCh37, Genome Reference Consortium Human Reference 37, February 2009).

#### Array-comparative genomic hybridization, microarray image, and data analysis

Digestion, labeling, hybridization, and data analysis of genomic DNA were performed according to the CytoChip Oligo Reference Manual (http://www.cytochip.com). In brief, sample and reference DNAs, 20 μg each, were fragmented, and 1.0–1.5 μg of the fragmented DNA was labeled using the Fluorescent Labeling System (dUTP) (BlueGnome). Labeled samples were purified, combined, and hybridized for 40 h at 65°C, 20 rpm, to the CytoChip Oligo slide (BlueGnome) against gender-matched reference DNAs. Then the arrays were washed and scanned using a High-Resolution Microarray Scanner (Agilent Technologies, Santa Clara, CA, USA). Array images were analyzed and data were extracted with Agilent’s Feature Extraction Software, which uses a Linear Lowess algorithm to obtain background-subtracted and normalized intensity values.

### Method for quantitative real-time polymerase chain reaction analysis

A total of 34 representative target sequences (RTSs) were selected for validation of the CNVR determined by the array-CGH analysis and of the regions 62 kb upstream and downstream of the CNVR. For each RTS, a pair of primers (Additional file 1: Table S1) was designed using the Primer 3 web tool (http://bioinfo.ut.ee/primer3-0.4.0/). In addition, the in silico polymerase chain reaction (PCR) program from the University of California Santa Cruz (UCSC) browser (http://genome.ucsc.edu) was used for specificity analysis to certify that the primer pair matched only the sequence of interest. The actin gene was co-amplified with the RTSs and served as an endogenous Ref. [4]. The relative comparative threshold cycle ($2^{{ - \Delta \Delta C_{t} }}$) method was used to quantify copy number changes, where ΔΔC_t_ = (C_t__RTS_ − C_t__Actin_) _Patient_ − (C_t__RTS_ − C_t__Actin_) _Healthy control_ [4, 5]. All qPCR reactions were run in triplicate using the ABI 7500 Real-Time PCR System (Applied Biosystems, Foster City, CA, USA). PCR amplifications were performed in a total volume of 20 μL consisting of 1 μL of genomic DNA (approximately 50 ng), 1 μL (20 pM/μL) each of forward and reverse primers, 10 μL of Master Mix (2×), and water (Roche Applied Science, Indianapolis, IN, USA) under conditions of 95°C for 5 min followed by 40 cycles of 95°C for 10 s and 60°C for 10 s. The average C_t_ value was calculated from three replications of each sample and normalized against the endogenous reference gene (*ACTB*) to give the ΔC_t_ value, from which $2^{{ - \Delta \Delta C_{t} }}$ was then calculated for each RTS. For autosomal chromosomes, a $2^{{ - \Delta \Delta C_{t} }}$ value around 1 indicated a normal copy number status for the RTS (without CNV), and a value around 1.5 indicated a triplicate CNV status.

### Method for gene content and functional annotation

The gene content within the CNVR was retrieved from the UCSC Genome Database (GRCh37, https://genome.ucsc.edu/) and the Ensembl genome browser 75 database (http://asia.ensembl.org/Homo_sapiens/Info/Index). The gene symbols retrieved were submitted to the DAVID resource [6] (http://david.abcc.ncifcrf.gov/summary.jsp), where Gene Ontology (GO) analysis [7] and Kyoto Encyclopedia of Genes and Genomes (KEGG) pathway analysis [8] were performed to determine functional enrichment for protein-coding genes within the CNVR.

## Results

### Array-comparative genomic hybridization and validation by quantitative real-time polymerase chain reaction

**Figure 2:**
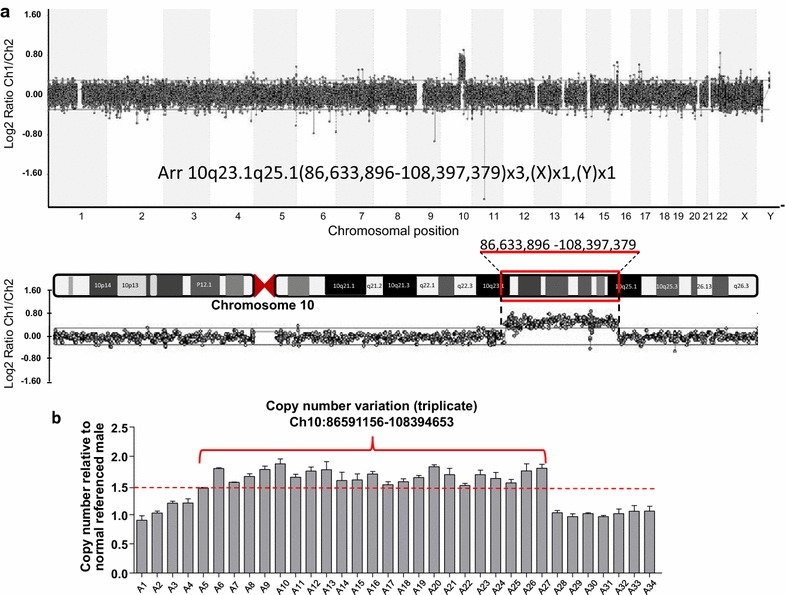
Array-comparative genomic hybridization and validation by quantitative real-time polymerase chain reaction analysis. **a** Array-comparative genomic hybridization analysis of patient genomic DNA vs. normal male genomic DNA. The characteristic duplication in the 10q23.1–10q25.1 region is clearly detectable in both the chromosomal segmentation analysis (*upper panel*) and the chromosome 10 profile (*bottom panel*). **b** The result of quantitative real-time polymerase chain reaction validation indicates that 23 successive representative target sequences representing the DNA sequence of Ch10:86591156–108394653 were confirmed in the triplicate copy number variation status (relative copy number ≥1.5).

In view of the clinical suspicion of the distal 10q trisomy syndrome, we performed genomic array-CGH analysis on the patient’s blood DNA sample referenced to a normal male control DNA sample. The result of our array-CGH analysis was arr 10q23.1q25.1 (86,633,896–108,397,379) × 3, (XY) × 1 (the International System for Human Cytogenetic Nomenclature, ISCN 2013), indicating that the patient had a 21.76 Mb CNVR appearing three times (trisomy) in the 10q23.1–10q25.1 region (Figure 2a). To address the limitation that the CytoChip Oligo array cannot detect DNA copy number changes in genomic regions that are not represented on the microarray, we complemented the array-CGH result with qPCR analysis, performing validation of the CNVR along with its 62 kb upstream and downstream regions. For this validation, 34 selected RTSs were tested for their CNV status relative to the normal reference genomic DNA. Of the 34 RTSs, 23 consecutive RTSs representing the Ch10:86591156-108394653 (21.8 Mb) region were confirmed in their triplicate copy number status (Additional file 1: Table S1; Figure 2b). These results support the conclusion that a 21.8 Mb duplication was present in chromosome region 10q23.1–10q25.1 of the patient.

### Gene content of copy number variation region

**Figure 3:**
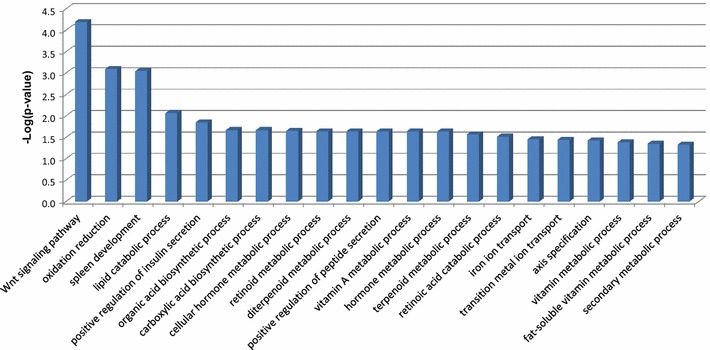
Gene Ontology analysis. A total of 200 protein-coding genes within the copy number variation region were subjected to Gene Ontology analysis and categorized by biological process. The logarithm was taken of the functional enrichment p-values by database for annotation, visualization and integrated discovery. A log (p-value) value around three indicates that the biological process is pertinent to the gene list.

For the chromosomal region encompassed by the CNVR, a total of 200 protein-coding genes, 25 pseudogenes, 10 microRNAs, 3 processed transcripts, 2 antisense RNAs, 1 miscellaneous RNA, 1 small nucleolar RNA, and 1 small nuclear RNA were retrieved from the UCSC and Ensembl genome databases (Additional file 1: Table S2). GO analysis for the 200 protein-coding genes showed a predominant representation of genes categorized by biological process (BP) under the terms of the Wnt signaling pathway, oxidation–reduction, and spleen development (Figure 3, Additional file 1: Table S3), genes categorized by cellular compartment (CC) under the term of centrosome (Additional file 1: Table S4), and genes categorized by molecular function (MF) under the terms of iron ion binding, oxygen binding, heme binding, and retinoid binding (Additional file 1: Table S5). KEGG pathway analysis revealed that these genes were by and large represented in metabolism-related pathways and the Wnt signaling pathway (Additional file 1: Table S6).

## Discussion and conclusions

In this report, our patient’s clinical presentation (Table 1) raised suspicion of the distal 10q trisomy syndrome, which prompted conventional cytogenetic testing, also known as karyotyping. However, this method revealed no detectable abnormal karyotype in this case. Conventional cytogenetic testing is known to have limitations due to poor chromosome morphology, inadequate metaphase yield, poor banding quality, and uniparental disomies [9–11], which may account for missed detection by this method in the present case. Therefore, array-CGH, a recommended first-line test in most cytogenetic departments [12, 13], was used to help delineate the chromosomal abnormalities of our patient, and it demonstrated a 21.76 Mb CNVR in chromosome region 10q23.1–10q25.1 (Figure 2a). Array-CGH has been shown to enable the genome-wide analysis of DNA copy numbers and allow screening of gains and losses of genetic material across the genome while offering the advantages of high resolution and high throughput [14].

**Table 1:** The common features of the distal 10q trisomy syndrome

Facial	Musculoskeletal	Other
Minor facial dysmorphism*	Microcephaly*	Growth retardation*
High/large forehead*	Hypotonia	Psychomotor retardation*
Fine eyebrows*	Joint laxity	Mental retardation*
Antimongoloid slants*	Camptodactyly*	Cardiac abnormalities
Ocular hypertelorism	Scoliosis	Ocular abnormalities
Epicanthic folds	Short neck	Renal abnormalities*
Flat nasal bridge*	Small bones and hands*	Brain abnormalities*
Short nose		Pulmonary defects
Low set ears		Autism spectrum disorder
Posteriorly rotated ears*		
Short palpebral fissures*		
Cleft palate		
Retrognathia		
Bow-shaped mouth*		

* Features present in the present report’s described case.

Despite the increased resolution and higher diagnostic yield associated with array-CGH, there may be a concern that this method alone is also likely to be insufficient for the exact mapping of a CNVR, as the set of oligonucleotide probes on a given microarray does not provide complete coverage of each chromosome, and some CNVs could be missed as a result. We reasoned that incorporating the additional CNVs detected by a different approach such as qPCR would result in a more precise determination of the size of the CNVR. Therefore, we performed qPCR analysis to complement the array-CGH result and were able to conclude the CNVR to be 21.8 Mb in size (Figure 2b). The above-mentioned limitation of array-CGH also explains the need to design the PCR primers for the 34 selected RTSs outside the boundaries of the microarray’s oligonucleotide probes in order to better confirm the CNVR. After comparing the CNVR identified in this study with the findings in previously published reports of distal 10q trisomy, we believe that the 21.8 Mb CNVR in the 10q23.1–10q25.1 chromosome region is a novel finding. Regarding the conceivable genetic mechanisms involved in the CNV of our patient, it is possible that one of the parents was a carrier of a balanced insertional translocation involving the CNVR, and the CNVR was subsequently transmitted to the offspring [15]. Ideally, a karyotype analysis of the parents should provide some genetic information. Unfortunately, karyotyping of the parents was precluded by their unique family culture, thereby making it difficult to ascertain the genetic condition involved in our patient’s distal 10q duplication.

The current case underlines the possible connection between clinical disorders and an aberrant biological process. The CNVR identified in this study covered a total of 200 protein-coding genes (Additional file 1: Table S2). The functions of some of these genes are classically annotated as the related genes of the Wnt signaling pathway (Additional file 1: Table S7), an evolutionarily conserved cellular signaling pathway that plays a crucial role in various biological processes such as organogenesis, tissue homeostasis, and pathogenesis of many human diseases [16]. Given the critical role the Wnt signaling pathway plays in early development, including a presumed role in brain [17] and kidney [18] development, deregulated Wnt signaling is expected to be involved in many kidney diseases, including renal cell carcinoma, Wilms’ tumor, renal fibrosis, cystic kidney disease, and acute renal failure [18–24]. Importantly, functional analysis of the genes within our patient’s CNVR using DAVID revealed the Wnt signaling pathway as the most relevant biological process (Additional file 1: Table S3). Hence, it is likely that an aberrant Wnt signaling pathway due to a copy number gain of related genes contributed to our patient’s significant clinical features including brain atrophy, stage II chronic kidney disease, and a cyst in an atrophic kidney (Figure 1). Additionally, it is tempting to speculate that the gain of an extra copy of the *WNT8B* and *LZTS2* genes was a causal factor for brain and kidney atrophy, respectively, in our patient. This hypothesis is supported by recent studies showing that aberrant early activation of the Wnt signaling pathway due to *WNT8B* overexpression resulted in defective brain development [25], and that a reduced level of nuclear β-catenin from *LZTS2* overexpression inhibited the transcriptional activity mediated by the Wnt/β-catenin signaling pathway and resulted in suppression of renal development [26]. Finally, it is interesting to speculate that the growth retardation and chronic kidney disease observed in the present case were related to the gain in copy number of the *PTEN* and *NFKB2* genes, respectively. The main function of PTEN is to suppress the PI3K/AKT pathway [27] which promotes cell proliferation [28]; thus, the presence of extra *PTEN* gene product may inhibit body growth. *NFKB2* gene duplication has been reported to correlate with fetal pyelectasis [29], which may result in nephrouropathies [30].

In conclusion, the findings presented in this report demonstrate the presence of a novel 21.8 Mb CNVR in the 10q23.1–10q25.1 chromosome region of a patient. This is also the first identification of the protein-coding genes within the CNVR and analysis of their biological meaning using DAVID. As suggested by the results of DAVID-based gene functional analyses, a gain in copy number of some Wnt signaling pathway–related genes most likely resulted in aberrant Wnt signaling activity and thereby contributed to our patient’s clinical outcomes including brain atrophy, bilateral renal atrophy with chronic kidney disease stage 2, and a simple renal cyst in the left kidney. In addition, our report may contribute to a better understanding of the genotype–phenotype correlation in the involvement of *PTEN* and *NFKB2* genes in growth retardation and chronic kidney disease. Thus, our findings have revealed the gene content in the duplicated region of 10q23.1–10q25 trisomy and may provide useful information for further investigation of associations between biological processes and important clinical features of distal 10q trisomy.

### Consent

Written informed consent was obtained from the patient’s parents for publication of this case report and any accompanying images. A copy of the written consent is available for review by the Editor-in-Chief of this journal.

## Additional files

Additional file 1: Detail information of the qPCR primers, gene content of the CNVR, and results of GO analysis.

## Abbreviations

array-CGH: array-comparative genomic hybridization
qPCR: quantitative real-time polymerase chain reaction
CNV: copy number variation
CNVR: copy number variation region
DAVID: database for annotation, visualization and integrated discovery
MS/MS: tandem mass spectrometry
MRI: magnetic resonance imaging
ISCA: International Standard Cytogenomic Array Consortium
PCR: polymerase chain reaction
UCSC: University of California Santa Cruz
GRCh37: Genome Reference Consortium Human Reference 37
RTS: representative target sequence
GO: Gene Ontology
KEGG: Kyoto Encyclopedia of Genes and Genomes
ISCN: International System for Human Cytogenetic Nomenclature.
